# Differential expression profiling of head and neck squamous cell carcinoma (HNSCC)

**DOI:** 10.1038/sj.bjc.6601373

**Published:** 2003-11-11

**Authors:** F Lemaire, R Millon, J Young, A Cromer, C Wasylyk, I Schultz, D Muller, P Marchal, C Zhao, D Melle, L Bracco, J Abecassis, B Wasylyk

**Affiliations:** 1 Institut de Génétique et de Biologie Moléculaire et Cellulaire, CNRS/INSERM/ULP, 1 Rue Laurent Fries, BP 10142, 67404 Illkirch cedex, France; 2 UPRES EA 34-30, Centre Paul Strauss, 3 rue de la Porte de l'Hôpital, 67085 Strasbourg, France; 3 Exonhit Therapeutics, 65 Boulevard Masséna, Paris F-75013, France

**Keywords:** hypopharynx, ‘unknown’ genes, functional classes, biomarkers, pharmaceutical targets, virtual Northern

## Abstract

Head and neck squamous cell carcinoma (HNSCC) is the fifth most common cancer in men with an incidence of about 780 000 new cases per year worldwide and a poor rate of survival. There is a need for a better understanding of HNSCC, for the development of rational targeted interventions and to define new prognostic or diagnostic markers. To address these needs, we performed a large-scale differential display comparison of hypopharyngeal HNSCCs against histologically normal tissue from the same patients. We have identified 70 genes that exhibit a striking difference in expression between tumours and normal tissues. There is only a limited overlap with other HNSCC gene expression studies that have used other techniques and more heterogeneous tumour samples. Our results provide new insights into the understanding of HNSCC. At the genome level, a series of differentially expressed genes cluster at 12p12–13 and 1q21, two hotspots of genome disruption. The known genes share functional relationships in keratinocyte differentiation, angiogenesis, immunology, detoxification, and cell surface receptors. Of particular interest are the 13 ‘unknown’ genes that exist only in EST, theoretical cDNA and protein databases, or as chromosomal locations. The differentially expressed genes that we have identified are potential new markers and therapeutic targets.

Head and neck squamous cell carcinoma (HNSCC) arises from the surface epithelium of the upper-aerodigestive tract (pharynx, hypopharynx, and larynx) and the oral cavity. Extensive epidemiological studies show that alcohol potentiates tobacco-related carcinogenesis and is also an independent risk factor. Head and neck squamous cell carcinoma is the fifth most common cancer in men with an incidence of about 780 000 new cases per year worldwide (Sankaranarayanan *et al*, 1998). Surgery and radiotherapy are highly effective in the treatment of stage I and II tumours, but over 70% of patients present with locoregionally advanced stage III or IV disease. Locoregional disease recurs in 60% of patients and metastatic disease develops in 15–25% (Genden *et al*, 2003). Furthermore, patients develop second primary tumours at an annual rate of 3–7% (Leon *et al*, 2002). However, less than 30% of HNSCC patients are free of disease after 3 years, and 5-year survival rates have remained largely unchanged in the last three decades (Dimery and Hong, 1993). The characterisation of the molecular determinants of the head and neck carcinogenesis process is essential for the better understanding of this malignancy and the development of rational targeted intervention.

Specific genes have been associated with the development or presentation of HNSCC, but these individual alterations have failed to define prognostic or diagnostic markers (reviewed in Leonard *et al* (1991) and Scully *et al* (2000)). Addressing this issue requires large-scale analysis of gene expression profiles. A number of recent studies have reported gene expression profiles of small numbers of HNSCC patients using commercial or focused microarrays (Leethanakul *et al*, 2000; Xie *et al*, 2000; Alevizos *et al*, 2001; Al Moustafa *et al*, 2002; Belbin *et al*, 2002; El-Naggar *et al*, 2002; Mendez *et al*, 2002). The microarray analysis is limited by the set of genes on the arrays, whereas polymerase chain reaction differential display (PCR-DD) randomly samples the transcriptome. The PCR-DD has been used to discover novel genes that would not have been identified using methodologies that cover a predefined range of genes (Glynne-Jones *et al*, 2001; Sasaki *et al*, 2001; Ying *et al*, 2001). We have performed the first randomised comparative analysis of gene expression of HNSCC patients using PCR-DD. We did not use microdissected tumour or normal components for this analysis since numerous studies have shown that the host tumour microenvironment influences tumour cells (van den Hooff, 1988; Nelson *et al*, 2000; Coussens *et al*, 1999; St Croix *et al*, 2000). We have identified a series of novel genes that exhibit striking differences in expression between HNSCC tumours and histologically normal matched tissues. They should contribute to a better understanding of HNSCC and provide new targets for therapeutics.

## MATERIALS AND METHODS

### Samples

Hypopharyngeal tumours and the corresponding histologically normal tissue, used with consent, were derived from surgical resections of squamous cell carcinoma. The patients had not been treated at the time of surgery, but were subsequently treated with radiotherapy. The samples used were resected near the advancing edge of the tumours avoiding their necrotic centres. They were comprised of 70–80% cancer cells in almost all cases, as assessed on adjacent histological stained sections. Normal samples were collected from the farthest margin of the surgical resections (usually uvula). The tumours were classified according to TNM stages (tumour, node, metastasis) based on the UICC criteria (Sobin and Wittekind, 1997), and grouped into three categories. The early (E) stage corresponds to small-sized tumours (T1/T2), moderately to well differentiated, without lymph node involvement. The two later-stage tumour types were of medium size (T2/T3), homogeneous differentiation and lymph node involvement (N1–N2c). At the time of resection, these later-stage tumours appeared clinically and histologically similar. However, during 3-year follow-up, one group of patients did not develop metastases (no metastatic propensity: NM), whereas the other developed metastases predominantly in the lung, bone, and liver (with metastatic propensity: M).

### PCR-Differential display

Total RNA was isolated with RNAeasy (Qiagen, Courtaboef, France), DNAseI treated, column purified (Qiagen) and pooled according to the tumour type (3 E, 2 NM and 2 M patients). The corresponding normal RNAs were similarly pooled. The PCR-DD was performed on the pooled samples using 58 5′ primers (HAP) in combination with three 3′ primers (HT11A/G/C) according to the GenHunter protocol and as described by Liang *et al* (Liang, 1998). All samples were prepared in duplicate from the reverse transcription stage to reduce experimental variability. Differential bands were isolated, reamplified with the corresponding primers, verified by agarose gel electrophoresis, and cloned in the pGEMt-Easy vector (Promega, Charbonnères, France). Eight colonies per band were expanded in liquid culture. A volume of 2 *μ*l of the cultures were used for PCR, in the same conditions as the reamplification, with the pGEME1 and pGEME2 primers (5′-CGC GGT ACC GGA TCC ATG CAT TGG CGG CCG CGG GAA TTC-3′ and 5′-CGC GGT ACC GGA TCC ATG CAT CAT ATG GTC GAC CTG CAG-3′, respectively). The fragments (50–800 base pairs) were verified by agarose gel electrophoresis, and subsequently the DNA was spotted directly onto nitrocellulose membranes (Hybond N+, Amersham, Les Ulis, France) using a 96-well vacuum-driven dot blot manifold (Bio-Rad, Marnes-la-Coquette, France). Filters underwent denaturation (1.5 M NaCl, 0.5 M NaOH) and neutralisation (1.5 M NaCl, 0.5 M Tris-HCl pH 7.2, 0.001 M EDTA) followed by UV cross-linking.

### Reverse Northerns

Owing to the limiting quantity of patient RNA, the SMART cDNA synthesis system (Clontech, Lee Pont de Claix, France) was used to reverse transcribe and amplify total RNA to be used as a probe. The first strand, synthesised from 0.2 *μ*g of total RNA, was amplified for a controlled number of cycles, to ensure linearity, as described by the manufacturer. The labelling was performed with 100 ng of SMART cDNA and a mix of the DD primers that originally generated the clones. The probes were purified through Sephadex G50 columns (Bio-Rad). The filters were hybridised in 10% dextran sulphate/0.1% SDS/10 mM NaCl overnight at 65°C, washed to a stringency of 0.2 × SSC/0.1% SDS at 65°C and exposed on Biomax film for 3–24 h at −80°C, and subsequently on Molecular Dynamics PhosphorImager screens (Orsay, France) for quantification on a Typhoon PhosphorImager analyser (Orsay, France). Positive clones were then expanded from the original liquid cultures and plasmid DNA extracted using standard alkaline lysis followed by purification through Nucleospin miniprep columns (Macherey-Nagel, Hoebdt, France). The sequences of the inserts were analysed with the BLAST algorithm at http://www.ncbi.nlm.nih.gov/bl ast/. Positive clones were then confirmed at least twice with probes generated (as above) from two independent SMART cDNA preparations. The filters included control positive clones that were systematically used for cross comparison.

### Classical Northerns

Total RNA was extracted from tissue samples with Trizol (Life Technologies, Cergy Pontoise, France). A measure of 20 *μ*g of RNA was subjected to agarose/6% formaldehyde gel electrophoresis, then transferred to Hybond N+ membranes (Amersham). [^32^P]-labelled probes were generated with the Rediprime system (Amersham). Membranes were prehybridised and hybridised in 50% formamide at 42°C according to the manufacturer's specifications, washed to a stringency of 0.1 × SSPE/0.1% SDS at 50°C and exposed to X-ray film (Kodak, Les Ulis, France). The level of expression in tumour samples was analysed in comparison with the matched normal tissues after correction for loading using RPLO. Ribosomal phosphoprotein P0 (RPLP0, originally called 36B4) is a ubiquitous expressed gene that has been routinely used in different laboratories as an internal control to normalise for the amount of RNA. In a large study (98 cases), we confirmed by real-time quantitative PCR (RT-QPCR) that its expression level remains relatively constant between HNSCC tumours and matched normal tissues (data not shown). RPLP0 gave better results than the commonly used control GAPDH, which was more variable between samples in our experiments.

### Virtual Northerns

A measure of 0.2 *μ*g of total RNA from individual patients was converted into SMART cDNA ((Franz *et al*, 1999) and the Clontech protocol). The optimal number of cycles for each sample was determined according to the manufacturer's instructions. Aliquots of the PCR products, after different numbers of cycles (15–25), were analysed by agarose gel electrophoresis and Northern blotting with RPLP0 as the probe. The amplification and the fidelity are considered to be optimum when the PCR is in the exponential phase of amplification, one or two cycles before reaching the plateau (range 17–20 cycles). The RPLP0 signal of the optimum PCR was used as an internal standard to equilibrate loading of the virtual Northerns. Appropriate amounts of this ‘SMART’ cDNAs were electrophoresed on agarose gels, transferred to Amersham Hybond N+ nylon filters. Probes were labelled with [^32^P]dCTP by random priming or PCR with the pGEME1 and pGEME2 primers (see above). Filters were hybridised in dextran sulphate (as above), exposed overnight to PhosphorImager screens and quantified using the Typhoon ImageQuant software. Filters were finally reprobed with RPLP0 to verify equal loading.

### Real-time quantitative PCR

RNA was quantitated with the LightCycler system (Roche Diagnostics, Meylan, France). A measure of 1 *μ*g of total RNA was reverse transcribed with random primers and the Superscript II RT–PCR system (Life Technologies). The PCR reactions were performed with the LC Fast start DNA master SYBR green I reaction mixture according to the manufacturer's instructions. Volumes of 2 *μ*l of 1 : 50 diluted RT products were used in 20 *μ*l reactions. The nucleotide sequences of the primers and their localisations are shown in Table 1

**Table 1:** Real-time quantitative PCR primers and reaction conditions.

**Clone**		**Primer**			**PCR**	
**Name**	**Acc. no.**	**Name**	**Sequence**	**Location**	**Size (bp)**	***T*** **(°C), [MgCl_2_] (mM), [NTP] (*μ*M)**
EMP1	NM_001423.1	EMP1-2F	GACCTCATGCCATGGTCTTT	1393–1412	237	62, 4, 0.5
		EMP1-2R	CTGCATTGAGGGGAATCCTA	1556–1575		
PIGR	AF272149.2	TB6-1F	CCACCGTGGAGATCAAGATT	1532–1551	186	62, 4, 0.5
		TB6-1R	CAGCCCGTGTTATTCCACTT	1669–1688		
PON2	NM_000305	AEY125-F	GCCAACAATGGGTCTGTTCT	993–1012	198	62, 4, 0.5
		AEY126-R	TGGGTCAATGTTGCTGGTTA	1171–1190		
Apol2	AF305225	AEY131-F	AGGCAGATGAGCTCCGTAAA	363–382	185	62, 4, 1
		AEY131-R	GACCTGCTCAACTCCTCTG	528–547		
DRG1	D87953	AEY139-F	GCTTTGGTCAGAGTGAATTGAA	2717–2736	182	62, 4, 0.5
		AEY140-R	CCGATCCCCGACTTTTCTAC	2879–2898		
PSMD8	NM_004159	AEY147-F	GAAGGAAGATGGTTGGGTA	981–998	189	62, 4, 0.5
		AEY147-R	TCTCTTTGGCTCAGGCTAGG	1150–1169		
RP1-68D18	BM285393	EAW253-F	TGCAAGTCACCACAACAGGT	153 589–153 608	185	62, 4, 0.5
		EAW254-R	AGCCTTGCATAAATGGCTGT	153 753–153 773		
HSPC150	NM_014176	AEY151-F	TGTTCTCAAATTGCCACCAA	390–409	191	62, 4, 1
		AEY152-R	TTGCATGCTTCTCTGTCCAC	516–580		
RPLP0	M17885	RPP0-3F	GAAGGCTGTGGTGCTGATGG	224–243	103	62, 3, 1.5
		RPLP0-R	CCGGATATGAGGCAGCAGTT	307–326		

Listed above are the clone names and accession numbers; the primer name, sequence (5′–3′) and location on the sequence associated with the accession number; and the PCR product size and reaction conditions (annealing temperature and concentrations of MgCl_2_ and NTP).

. The primers were chosen with the Primer3 software and their specificity was verified by BLAST analysis on the nr database (non redundant set of GenBank, EMBL, and DDJB databases). For each gene, a standard curve was constructed using serial dilutions of a single standard cDNA (equivalent to 100, 40, 20, 10, 4, 2, and 1 ng of total RNA) derived from a pool of 10 hypopharyngeal tumours. The concentrations of primers, MgCl_2_, probes, and cDNA were optimised to obtain linear standard curves. Unknown samples were estimated relative to these standard curves. For genes overexpressed in tumours, expression levels were calculated relative to the median values for normal tissue, and *vice versa* for genes expressed at higher levels in normal tissues. PCR reactions were run at least twice for each sample. The mean value was retained whenever the standard deviation did not exceed 15%, and normalised using RPLP0 as an internal control.

## RESULTS

### PCR differential display

A large-scale PCR-DD was performed on patient RNA derived from three stages of HNSCC (Table 2

**Table 2:** Characteristics of the tumours

**Patient**	**Tumour**	**T**	**N**	**M**	**Diff**	**Sex**	**Age (years)**	**Treatment after surgery**	**Evolution**	**Disease-free survival (months)**	**Overall survival (months)**	**Actual state**
1	E	2	0	0	2	M	53	RX	0	11	15	D
2	E	1	0	0	2	M	61	N	0	15	25	D
3	E	2	0	0	2	M	44	N	0	41	41	A
4	NM	3	2b	0	3	M	66	RX	0	54	58	D
5	NM	3	2b	0	2	M	52	RX	SC	39	44	A
6	M	2	2c	0	3	M	54	RX	M	16	25	D
7	M	3	2b	0	2	M	54	RX	M	5	7	D

**Figure 1:**
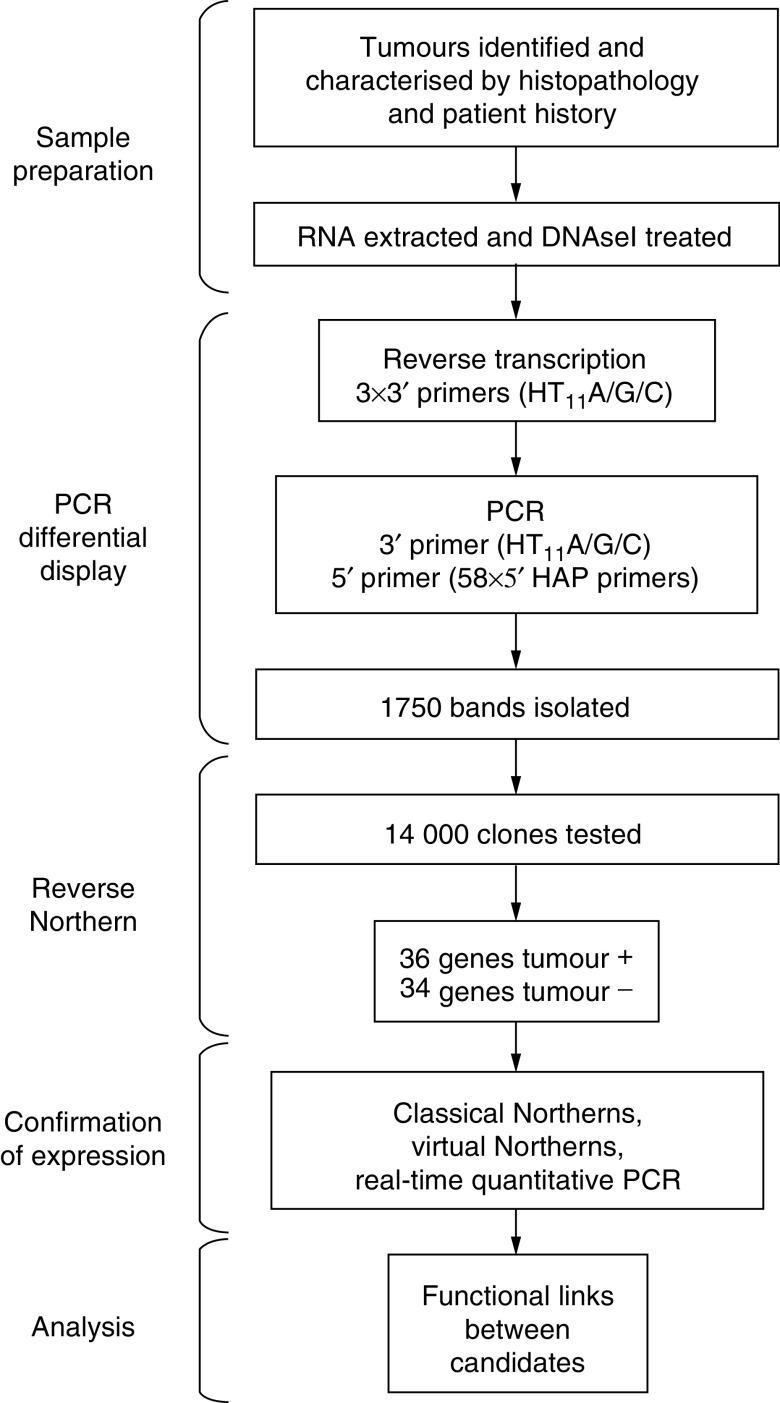
Flowchart outlining the study. The flowchart indicates how the tumour samples were selected and processed, the PCR-DD primers that were used and the number of bands isolated, the number of clones tested by reverse Northern, the resulting number of genes identified, the types of confirmation used to validate the results, and the bioinformatics analysis to analyse the results.

**Figure 2:**
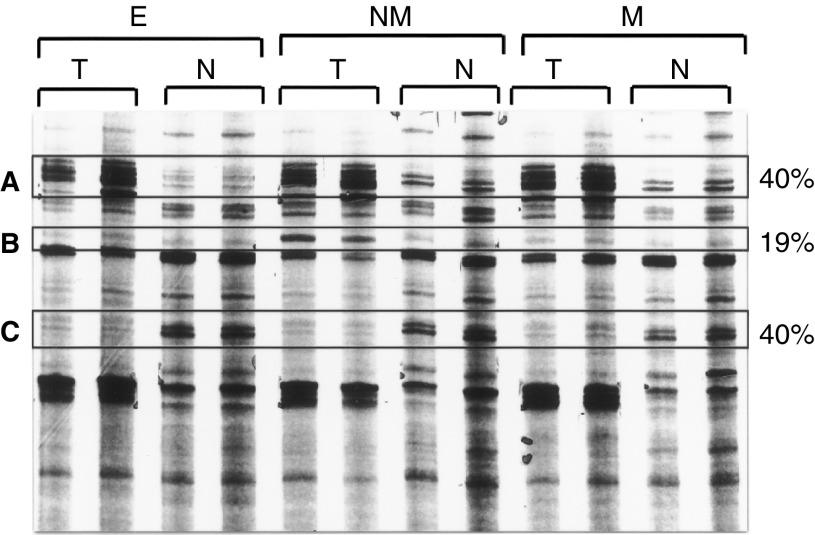
Differential display gel comparing the three stages of tumours (T) with their corresponding normal (N) samples. E=early; NM=no metastatic potential; M=metastatic propensity. Highlighted are the three types of profiles (**A**, overexpressed in tumour; **B**, tumour-specific profiles; **C**, underexpressed in tumour), and the percentages give the overall proportions in these categories.

All the tumours were localised in the hypopharynx. T, N, and M correspond to the TNM nomenclature for tumour stage (tumour, node, and metastasis). *Diff (differentiation)*: 1=well, 2=moderate, 3=poorly. *Treatment after surgery*: RX=radiotherapy; N=no treatment. *Evolution*: 0=no evolution, M=metastasis, SC=secondary cancer. *Actual state*: D=dead, A=alive.

) and corresponding normal tissues (see Figure 1

for a methodology outline). Three 3′ primers (HT11A/G/C) and 58 5′ primers (HAP1-10, 33–80) were combined to cover theoretically over 90% of expressed sequences (Liang, 1998). This experimental design maximises the detection of ‘novel’ sequences, a strength of PCR-DD compared to DNA arrays. Around 95% of the bands showed no difference in signal intensity across the different samples, as expected. Of the 1750 bands that did show a difference, 40% were increased and another 40% were diminished in all the tumour samples (Figure 2

). These two groups are the focus of this initial study. The 19% of the bands that differed between the tumour types will be addressed in future studies. Less than 1% of the bands differed in intensity between the normal samples from different patients, indicating that the differences observed between the normal and tumour samples were due specifically to the development of the tumours and not due to either patient polymorphism or PCR-derived artefacts (Figure 2). The differential bands were isolated and cloned, and eight clones were taken from each band for further analysis.

### Identification of the genes

**Figure 3:**
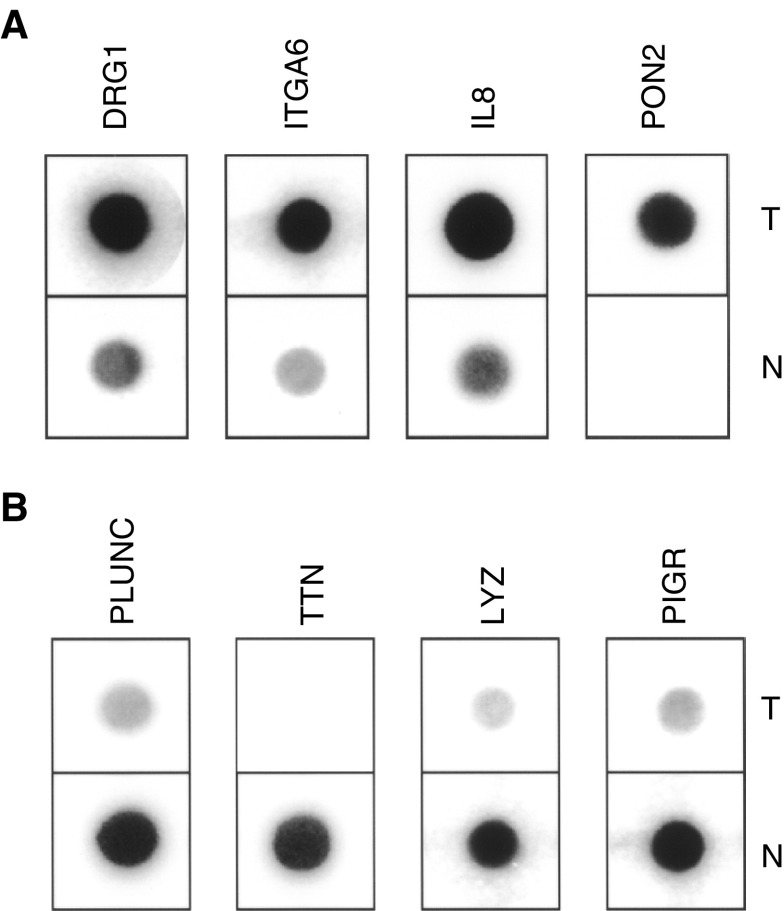
Reverse Northerns using tumour (T) or normal (N) tissue probes. The genes shown, which are overexpressed (**A**) or underexpressed (**B**) in tumours, are the first four in Tables 3A and B, respectively.

Reverse Northern hybridisation (Zhang *et al*, 1997; Trenkle *et al*, 1999) was performed on the 14 000 clones resulting from the DD to determine which clones among the eight clones derived from each band contained differentially expressed sequences. Macroarrays of the clones were hybridised with probes derived from either pooled tumour or pooled normal RNA, and the resulting signals were quantified. In total, 2500 clones presenting a tumour/normal signal ratio of >2.0 or <0.5 were grouped onto secondary arrays and reprobed twice for confirmation (Figure 3

). Clones with consistently differential profiles after multiple hybridisations and tumour/normal ratio of >2.0 (2–5-fold) or <0.5 (0.5–0.07-fold) were sequenced and identified using the BLAST algorithm. Some of the clones with consistent profiles corresponded to the same gene (1–85 clones per gene). Our final list contains 36 genes that are overexpressed in tumours (Table 3A

**Table 3:** Differentially expressed genes identified by PCR differential display (PCR-DD) and reverse Northern analysis in Parts A and B

**Order**	**Clone**	**Gene**	**Chromosome**	**Unigene or acc. no.**	**Immune**
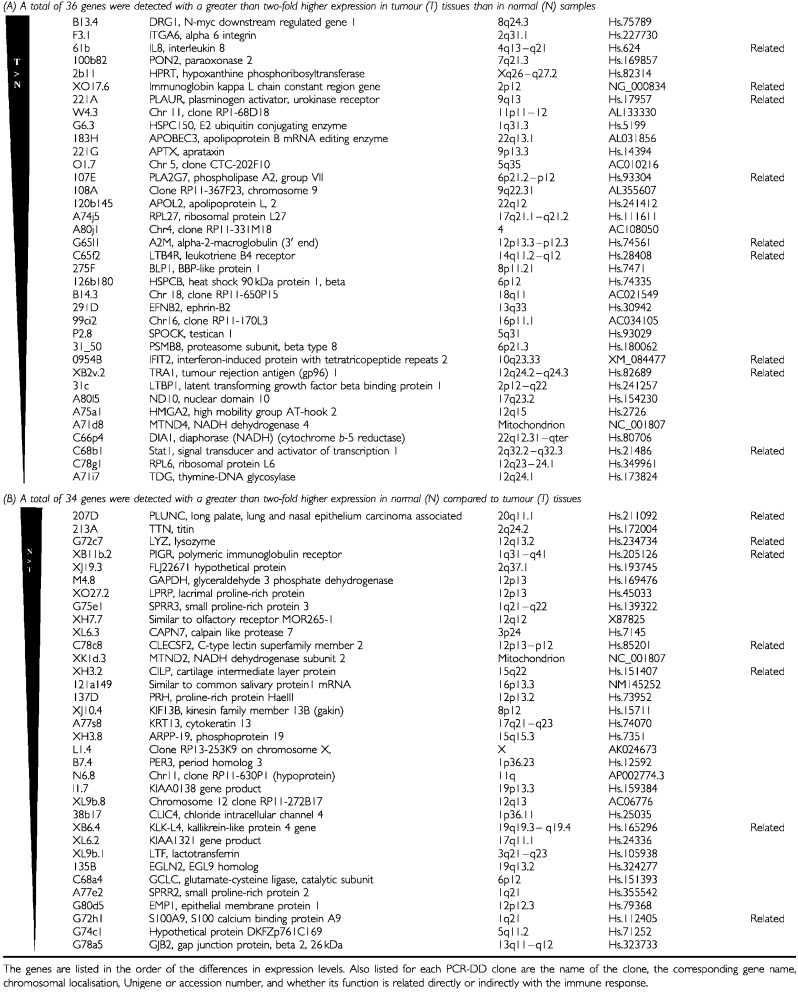					

The genes are listed in the order of the differences in expression levels. Also listed for each PCR-DD clone are the name of the clone, the corresponding gene name, chromosomal localisation, Unigene or accession number, and whether its function is related directly or indirectly with the immune response.

) and 34 genes that are under expressed (Table 3B). Six of the overexpressed and seven of the underexpressed sequences are novel, in that they do not correspond to known genes.

### Validation of gene expression profiles

**Figure 4:**
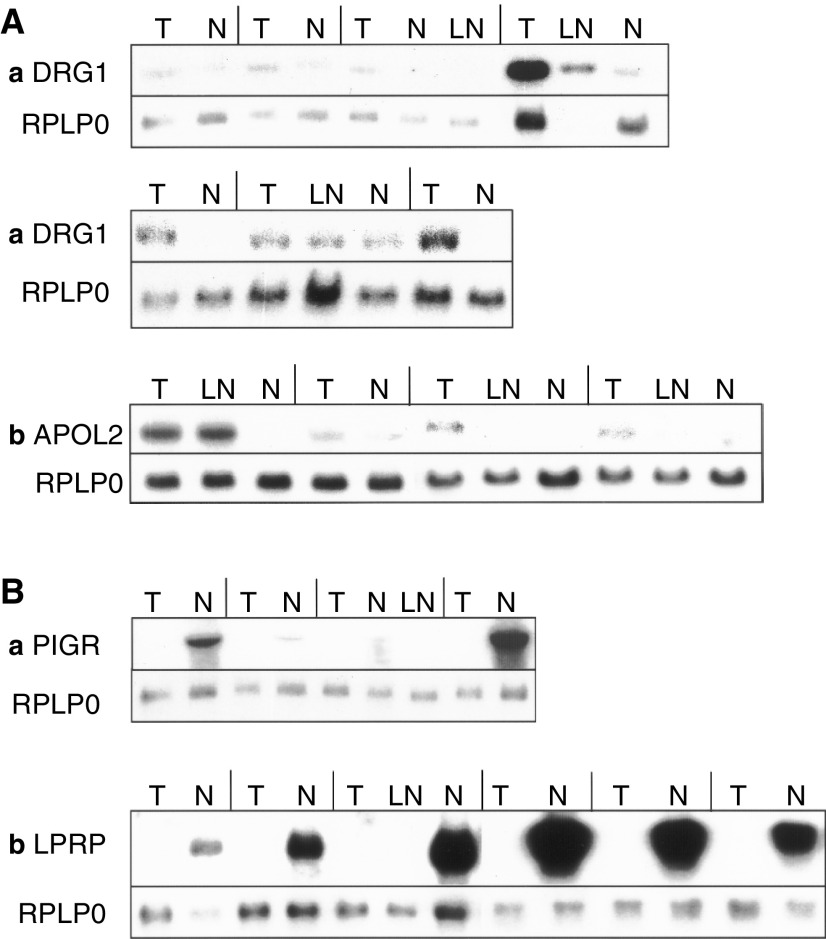
Classical Northerns: tumour (T), lymph node (LN) and normal (N) samples from the same patients were analysed. The RPLP0 control is shown under each lane. (**A**) Genes overexpressed in tumours: (a) DRG1, (b) APOL2. (**B**) Genes underexpressed in tumours: (a) PIGR, (b) LPRP. The lines separate the samples from particular patients, and comparisons should be made between the samples from each patient.

**Figure 5:**
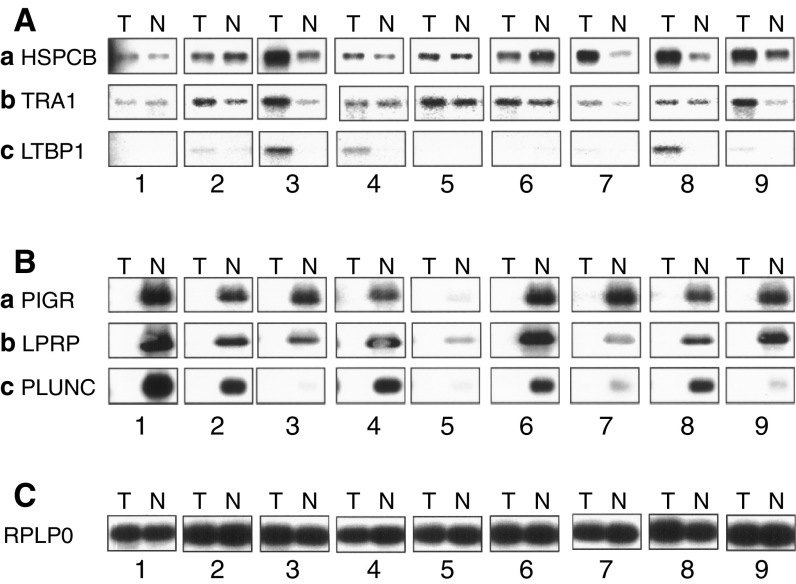
Virtual Northerns: the lanes 1–3 (E), 6,7 (NM) and 8,9 correspond to individual patients who were pooled for the PCR-DD. Tumour (T) and normal (N) samples from the same patient were compared. (**A**) Genes overexpressed in tumours: (a) HSPCB, (b) TRA1, and (c) LTBP1. (**B**) Genes underexpressed in tumours: (a) PIGR, (b) LPRP, and (c) PLUNC. (**C**) RPLP0 is the internal control.

**Figure 6:**
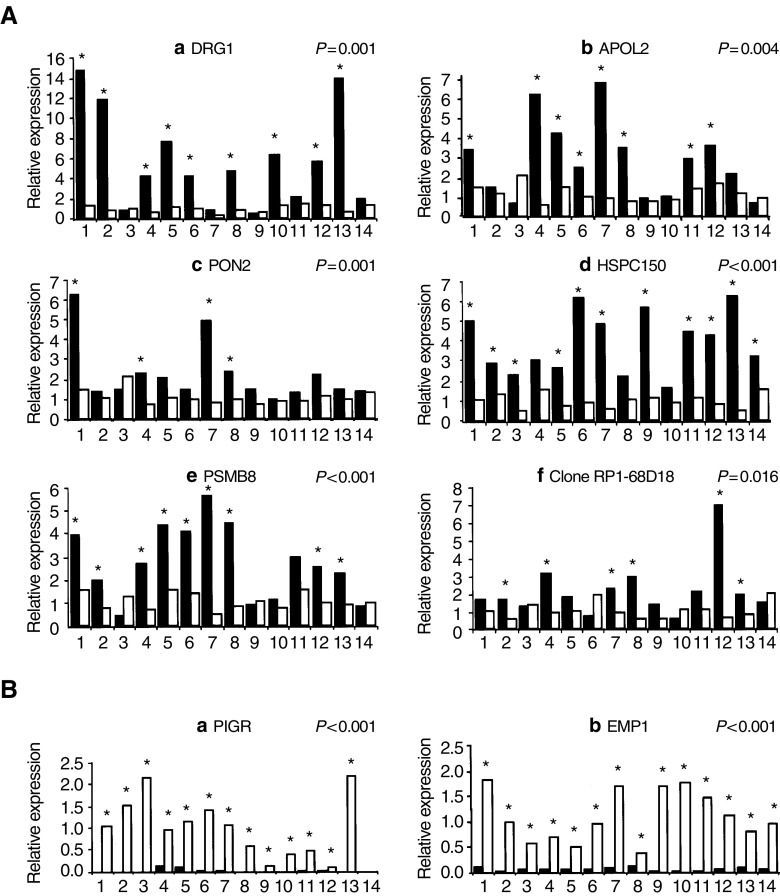
Real-time quantitative PCR. (**A**) Genes overexpressed in tumours (T): (a) DRG1, (b) APOL2, (c) PON2, (d) HSPC150, (e) PSMB8, and (f) RP1-68D18. (**B**) Genes underexpressed in tumours: (a) PIGR and (b) EMP1. The values for the tumours (black columns) and matched normal (N) tissue (white columns) were adjusted according to RPLP0, the internal control. The median of the N values was set to 1. The patients indicated with a star have a matched T/N ratio greater or equal to 2. The *P*-values of *t*-tests between the tumour and normal tissues are indicated.

To confirm that the large-scale analysis had correctly identified differentially expressed sequences, some up- and downregulated genes were analysed by the classical Northern analysis (Figure 4

). As the amount of patient material was too limited to do numerous classical Northerns, SMART technology (Clontech) was used to generate virtual Northerns (Figure 5

). In addition, RT-QPCR was used with a panel of 14 hypopharyngeal carcinomas and matched normal tissues (Figure 6

). The results were consistent across these validation techniques (DRG1, Figures 4Aa and 6Aa; APOL2, Figures 4Ab and 6Ab; PIGR, Figures 4Ba, 5Ba, and 6Ba; LPRP, Figures 4Bb and 5Bb; note that the patients were different). We found that PIGR, LPRP, PLUNC, and EMP1 are downregulated in almost all the tumours (Figures 4B, 5B, and 6B). DRG1, APOL2, HSPCB, TRA1, LTBP1, PON2, HSPC150, PSMB8, and clone RP1-68D18 are overexpressed in tumours at various frequencies (Figures 4A, 5A, and 6A). The differences in expression, measured by RT-QPCR, were at least two-fold in at least half of the tumours for seven of the eight genes analysed. DRG1, APOL2, HSCPC150, and PSMB8, and the novel sequence clone RPI-68D18 are overexpressed in nine, eight, 12, eight, and six patients, respectively. Overall, the expression profiles correlate well with the behaviour observed at the DD band level.

## DISCUSSION

We have compared the expression profiles of hypopharyngeal tumours with matched normal tissues by the PCR-DD. This study of a specific site of HNSCC provides a novel collection of cancer-related genes. Our results are of high quality since the DD sequences were reselected with several rounds of reverse Northerns, and there was a consistent correlation between the DD profiles and analyses by classical Northerns, virtual Northerns, and RT-QPCR. The sequences reported here had a consistent DD profile across the tumour samples, whereas other bands (about 20%) with tumour stage-specific profiles need to be studied further with a larger number of tumours. Only eight out of 70 genes overlap between our and other profiles of HNSCC (Leethanakul *et al*, 2000; Alevizos *et al*, 2001; Al Moustafa *et al*, 2002; Belbin *et al*, 2002; El-Naggar *et al*, 2002; Mendez *et al*, 2002; Xie *et al*, 2000), possibly because, in contrast to these other studies, we did not restrict the profiling to particular genes on arrays, since PCR-DD samples the whole transcriptome. Moreover, we restricted our analysis to a very specific site. Six of the common genes are expressed in the same manner (ITGA6, PON2, STAT1, KRT13, SPR2, and EMP1). In contrast to these studies, we found that GJB2 is underexpressed in tumours and DRG1 is overexpressed. In our experiments, DRG1 was shown to be overexpressed by four techniques (DD-PCR, reverse Northerns, classical Northerns, and RT-QPCR). Furthermore, DRG1 has been shown to be overexpressed in other tumours (see Cangul *et al*, 2002). There is some overlap between our list and profiles of other cancers (see Table 4

**Table 4:** Comparison with other tumours

**Order**	**Gene**	**Cancer**	**Expression**	**Function**	**Ref**
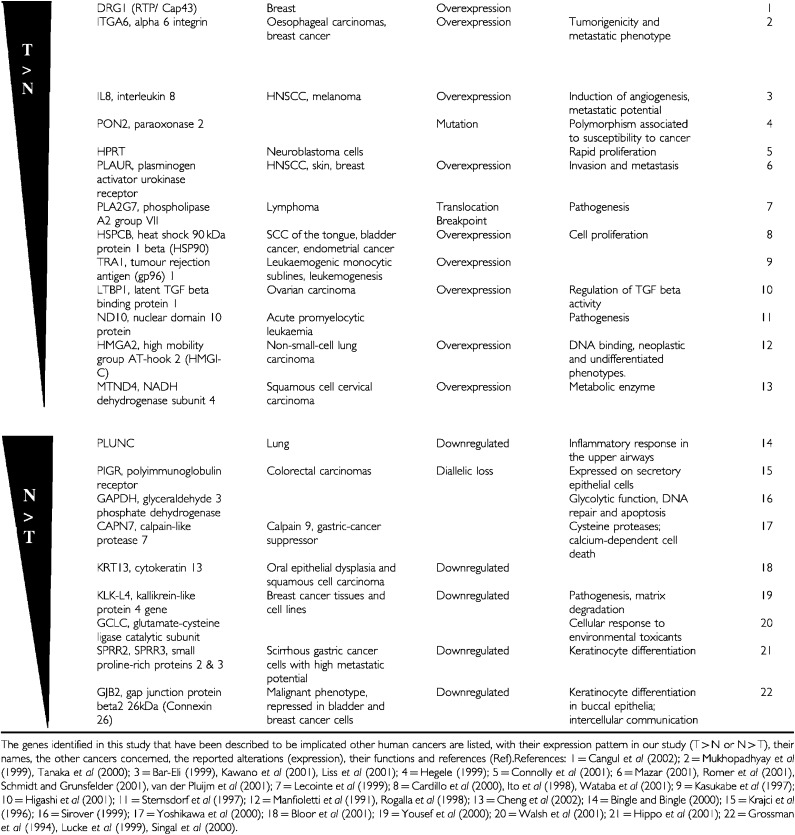					

The genes identified in this study that have been described to be implicated other human cancers are listed, with their expression pattern in our study (T>N or N>T), their names, the other cancers concerned, the reported alterations (expression), their functions and references (Ref).References: 1=Cangul *et al* (2002); 2=Mukhopadhyay *et al* (1999), Tanaka *et al* (2000); 3=Bar-Eli (1999), Kawano *et al* (2001), Liss *et al* (2001); 4=Hegele (1999); 5=Connolly *et al* (2001); 6=Mazar (2001), Romer *et al* (2001), Schmidt and Grunsfelder (2001), van der Pluijm *et al* (2001); 7=Lecointe *et al* (1999); 8=Cardillo *et al* (2000), Ito *et al* (1998), Wataba *et al* (2001); 9=Kasukabe *et al* (1997); 10=Higashi *et al* (2001); 11=Sternsdorf *et al* (1997); 12=Manfioletti *et al* (1991), Rogalla *et al* (1998); 13=Cheng *et al* (2002); 14=Bingle and Bingle (2000); 15=Krajci *et al* (1996); 16=Sirover (1999); 17=Yoshikawa *et al* (2000); 18=Bloor *et al* (2001); 19=Yousef *et al* (2000); 20=Walsh *et al* (2001); 21=Hippo *et al* (2001); 22=Grossman *et al* (1994), Lucke *et al* (1999), Singal *et al* (2000).

), which potentially identifies genes with general functions in cancer.

The genes we have identified have a biased chromosomal distribution, with many located at 12p12–13 and 1q21–22 (Table 3). Out of 70 genes, six localise to 12p12–13 (A2M, GAPDH, LPRP, CLECSF2, PRH, and EMP1), and three to 1q21–22 (SPRR3, SPRR2, and S100A9). These are the two most frequently altered regions in nasopharyngeal carcinoma (Marenholz *et al*, 1996; Chen *et al*, 1999; Salomon-Nguyen *et al*, 2000; Sato *et al*, 2001), indicating that transformation has complex effects on epidermoid cell biology.

We identified sequences that might be expressed in non epidermoid cells in the tumours, including endothelial-specific and immune-related genes. EFNB2, which is overexpressed in tumours, is a trans-membrane ligand specifically expressed in arterial endothelial cells (Gale *et al*, 2001). Of the 57 known genes, 16 are immune related (Table 3), and, in particular, the nine that are overexpressed could be considered as potential circulating markers for diagnostic purposes. Certain of the immune-related genes have also been associated with epithelial tissue differentiation and growth control, including PLUNC (Iwao *et al*, 2001), PIGR (Nihei *et al*, 1996), Stat1 (Maziere *et al*, 2000), and HSPCB (Edwards *et al*, 1991), PLAUR (Chapman and Wei, 2001; Ahmed *et al*, 2002) and PLA2G7 (Tao *et al*, 1996). In particular, PLAUR is a pan T cell activating antigen that has also been associated with epithelial-derived tumour development. It interacts with integrins to regulate cell–matrix interactions (Chapman and Wei, 2001; Ahmed *et al*, 2002). PLAUR and PLA2G7 are linked, as PLAUR is activated by PAF, which in turn is a substrate of PLA2G7 (Tao *et al*, 1996).

Some of the differentially expressed genes are involved in detoxification pathways and cellular defences against insults. Physiological response to environmental insult from tobacco and alcohol is particularly important in HNSCC (Johnson, 2001) and the differential expression of xenobiotic and detoxification enzymes has been reported in other transcriptome level studies (Alevizos *et al*, 2001). We identified two genes involved in antioxidation, GCLC (Talalay, 2000) and PON2 (Ng *et al*, 2001), and another involved in the response to oxidative damage to DNA, TDG (Laval, 1996). Cellular defences against insults could also account for the overexpression of heat shock and stress proteins, such as HSPCB, TRA1 (Maki *et al*, 1990) and DRG1 (Agarwala *et al*, 2000).

Cell-surface receptors, membrane-associated proteins and enzymes that are overexpressed in tumours are potential tumour markers and targets for drug design (Nam and Parang, 2003). We identified four overexpressed cell surface and membrane associated proteins (ITGA6, GJB2, PLAUR, and EFNB2) and nine enzymes (PON2, HPRT, HSCP150, APOBEC3, PLA2G7, HSPCB, MTND4, DIA1, and TDG). Interestingly, inhibitors of HSPCB are currently being tested in clinical trials (Neckers, 2002).

The major strength of the PCR-DD is to identify unknown genes from limiting amounts of biological material. We identified 13 differentially expressed sequences that exist only in the EST, theoretical cDNA or hypothetical protein databases, or correspond to chromosomal locations. One of these, clone RPI-68D18, was confirmed to be overexpressed in tumours by RT-QPCR. This sequence is homologous to a number of ESTs but otherwise has no significant relationship to cDNAs or proteins in the GENEMBL databases. The differences in expression we report provide insights into the biology of HNSCC and subjects for further study. The gene products that are expressed on the cell surface or have enzymatic activity are particularly noteworthy, since successful therapeutics have been developed against these types of molecules. Finally, the novel sequences may open totally new avenues for further research and development of new therapeutics and markers.
